# Bidirectional Associations of Adolescents’ Momentary Social Media Use and Negative Emotions

**DOI:** 10.1007/s42761-024-00244-2

**Published:** 2024-06-26

**Authors:** Tyler Colasante, Katie Faulkner, Dana Kharbotli, Tina Malti, Tom Hollenstein

**Affiliations:** 1 Humboldt Science Center for Child Development (HumanKind), Faculty of Education, Leipzig University, Leipzig, Saxony Germany; 2 Department of Psychology, Queen’s University, Kingston, Ontario Canada; 3 Centre for Child Development, Mental Health, and Policy, University of Toronto Mississauga, Mississauga, Ontario Canada

**Keywords:** Social media, Negative emotions, Mental health, Adolescence, Experience sampling

## Abstract

Public discourse and empirical studies have predominantly focused on the negative repercussions of social media on adolescents’ mental health. However, pervasive social media use is a relatively new phenomenon—its apparent harms have been widely accepted before sufficient longitudinal and experimental research has been conducted. The present study used an intensive longitudinal design (four assessments/day × 14 days; *N* = 154 12- to 15-year-olds (*M*_age_ = 13.47, *SD* = 0.58); *N* = 6,240 valid measurement occasions) to test the directionality of social media–negative emotion links in early adolescence, accounting for the type of social media usage (i.e., browsing vs. posting). The significance of effects depended on social media type: browsing predicted higher-than-usual negative emotions hours later, whereas no significant directional effects emerged for posting. The browsing effect was small but held after controlling for prior levels of negative emotions. It did not replicate concurrently, underscoring the importance of process-oriented designs with mental health symptoms tested shortly *after* passive social media usage. The results partially support the active-passive hypothesis, which singles out passively engaging with others’ curated social media content as most detrimental to mental health. Nonetheless, the small browsing effect and overall null-leaning pattern of effects imply that mediators and moderators are needed to further understand when using social media is problematic, beneficial, or neither.

**Supplementary Information:**

The online version contains supplementary material available at 10.1007/s42761-024-00244-2.

Social media is currently theorized as detrimental to adolescents’ mental health, with suspected mechanisms including social comparison, isolation, and disruption of routines, among others (Schønning et al., 2020). However, empirical research in this area is still at a relatively early stage. As highlighted in a recent US Surgeon General’s Advisory (Office of the Surgeon General, 2023), three fundamental research gaps limit our current understanding of the social media–mental health link in adolescence: First, more longitudinal studies are needed to discern whether social media use precedes or follows adolescents’ mental health symptoms. Second, the type of social media usage is rarely differentiated in longitudinal analyses despite theory and some studies suggesting that passive usage (i.e., browsing) is more strongly associated with poor mental health than active usage (e.g., posting; Frison & Eggermont, 2017; Thorisdottir et al., 2019). Third, studies on the role of social media in mental health are relatively rare during early adolescence, a sensitive period of emerging social media use, identity formation, and greater susceptibility to social pressures (Allison & Schultz, 2001; Spies Shapiro & Margolin, 2014). Focusing on momentary negative emotions as a dimension of mental health, the present study aimed to address these gaps by assessing temporal associations between active and passive social media use and negative emotions in a sample of early adolescents.

## Gap 1: Limited Longitudinal Studies Assessing Bidirectional Links Between Adolescents’ Social Media Use and Mental Health

### Long-Term Longitudinal Evidence

Here, we review the current state of longitudinal research testing the directionality of social media–mental health links, beginning with long-term studies spanning months and years. Theoretically, establishing directionality is an important step for understanding the causal relationship between variables because it helps inform the “flow of influence” (Hamaker & Wichers, 2017; e.g., whether social media usage typically precedes negative emotions or follows them). Raudsepp (2019) found that problematic social media use at age 15 years was associated with increased depressive symptoms from age 15 to 16. Moreover, social media use positively predicted subsequent depression, physical anxiety, and social anxiety in a large sample of Icelandic adolescents (*N* = 12,114; Thorisdottir et al., 2020). However, a longitudinal study of within-person effects across 8 years of adolescence found that engaging in higher levels of social media in a given year did not predict higher levels of depressive symptoms the following year (Coyne et al., 2020). Null effects of social media usage on subsequent depression were also found in a study of two cohorts with annual assessments spanning 2 and 6 years, respectively (Heffer et al., 2019). Null effects of social media usage have also been documented for adolescents’ subsequent anxiety (Jensen et al., 2019), overall well-being (Schemer et al., 2021), and generalized mental health (Beeres et al., 2021). Some evidence exists for the alternative directional effect of mental health predicting subsequent social media usage, but only in girls (Heffer et al., 2019) or to a small degree (Kelly et al., 2022). Further, with their robust bidirectional panel design, Coyne and colleagues (2020) found no significant effects of mental health symptoms on subsequent social media usage.

Overall, long-term longitudinal studies have produced small-to-null effects. Moreover, the directional focus of such studies on social media usage predicting subsequent mental health—as opposed to mental health predicting subsequent social media usage—increases the risk of confirmation bias for the few effects detected.

### Short-Term Longitudinal Evidence

Months- or years-long lags of time between measurements of social media use and mental health do not align with extant theorizing on how these constructs are related. Moreover, longitudinal studies with large lags are more susceptible to interim confounding effects, making it difficult to strictly assess the theorized relation of interest (Gollob & Reichardt, 1987). The social media–mental health link is typically regarded as a moment-to-moment process, with negative emotions occurring alongside or in the hours after social media use (see Schønning et al., 2020). Such momentary processes can be captured with the experience sampling method, an intensive, short-term longitudinal approach that involves asking participants to repeatedly report on their naturally unfolding thoughts, feelings, and behaviors.

A growing number of experience sampling studies have been conducted on this topic (e.g., Beyens et al., 2020; James et al., 2023; Jensen et al., 2019; Nereim et al., 2022), with most focused on momentary affect as a proxy of mental health/well-being. The vast majority of these studies assessed concurrent associations and yielded null results. For instance, Jensen and colleagues’ (2019) daily diary study found that adolescents’ negative affect (e.g., sadness, worry) was not worse on days when they spent more time on social media. Measuring multiple moments per day, Beyens et al. (2020) found no overall association between adolescents’ social media use and happiness within the same moments.

Only a few experience sampling studies identified in this review assessed the directional flow of influence (i.e., the temporal component of causality) by lagging their respective outcome variables. In a 14-day study with five measures per day, Kross and colleagues (2013) found that higher Facebook usage at one prompt predicted higher negative emotionality and lower subsequent well-being at the next prompt. However, similar experience sampling studies with six measures per day conducted by Beyens et al. (2021) and Valkenburg, Beyens et al. (2022) found no clear associations between adolescents’ social media usage and subsequent well-being. Given these mixed findings, scholars have argued for distinguishing between passive and active types of social media usage to gain a clearer understanding of implications for adolescent mental health (e.g., Schmuck et al., 2019).

## Gap 2: Limited Differentiation of Passive vs. Active Social Media Use in Longitudinal Studies

Passive social media use involves browsing others’ content without directly responding to or commenting on it. Active use involves directly engaging with others on social media by creating and posting content and/or replying to others’ content (Thorisdottir et al., 2019). According to the active-passive hypothesis, passively absorbing others’ curated content may increase one’s risk of experiencing envy, low self-esteem, and downstream mental health issues, whereas active social media use may boost opportunities for social engagement, support, and related positive emotions (Schmuck et al., 2019). Cross-sectional evidence for this distinction has been mixed (for a review, see Valkenburg, van Driel et al., 2022) and corresponding longitudinal tests have been sparse.

Some long-term longitudinal evidence aligns with the active-passive hypothesis. One study linked browsing to later declines in well-being, but the sample consisted primarily of adult Facebook users (*M*_age_ = 48 years; Shakya & Christakis, 2017). Puukko and colleagues (2020) found that depressive symptoms were linked to increases in posting, suggesting posting as a potential coping response. Frison and Eggermont (2017) found that elevated browsing predicted heightened depressive symptoms 6 months later, whereas elevated depressive symptoms predicted greater subsequent posting behaviors. In contrast, some studies found no clear effects of passive and active usage on subsequent mental health (Booker et al., 2018; Fredrick et al., 2022; Steinsbekk et al., 2023; Wang et al., 2019), nor of mental health on subsequent passive and active usage (Steinsbekk et al., 2023).

Only two identified short-term longitudinal studies assessed the direct, lagged associations of adolescents’ passive and active social media usage and mental health. Deploying six prompts per day over 21 days (*N* = 32,755 assessments), Valkenburg, Beyens et al. (2022) found that 20% of 13- to 15-year-olds displayed the theorized relation between passive use and lower affective well-being, whereas 80% did not. Using the same dataset, Beyens and colleagues (2021) found that only one of 387 participating adolescents fully conformed with the active-passive hypothesis by reporting a negative effect of passive use and a positive effect of active use. These studies challenge the ubiquity of the active-passive hypothesis while fulfilling the design features argued for here (i.e., intensive short-term measurement, lagging). However, both of them using the same dataset warrant further testing of the active-passive hypothesis with different samples.

## Gap 3: Limited Focus on Early Adolescence

Of all the pertinent studies reviewed here, less than one-third focused exclusively on early adolescence. Early adolescence is a formative period marked by the onset of puberty and corresponding changes in the limbic system that increase sensitivity to reward, novelty, and peers (Blakemore and Mills, 2014; Steinberg & Monahan, 2007; Sturman & Moghaddam, 2012). Young adolescents are also just beginning the process of forming their identities in relation to others (Allison & Schultz, 2001), and for recent generations, social media has become an inseparable part of this journey (Spies Shapiro & Margolin, 2014). An analysis of 84,011 participants ranging from 10 to 80 years of age found the strongest negative links between social media use and life satisfaction in early adolescence (Orben et al., 2022). In addition to experiencing the biological and psychological changes of pubertal onset, young adolescents may have fewer tools to navigate drama on social media (Lenhart, 2015), a less established sense of self and social circle, both offline (Meeus, 2011) and online (Lenhart, 2015), and worse emotion regulation skills (Hollenstein & Lougheed, 2013) than their older adolescent counterparts. As such, younger adolescents may be more susceptible to the negative emotional effects that arise while communicating with peers and portraying one’s identity in the socially demanding arena of social media (also see Orben et al., 2022).

## The Present Study

To address the aforementioned gaps, the present study tested momentary bidirectional associations of active and passive social media use and negative emotions in early adolescence. Due to scant short-term longitudinal studies, none of which fully tested bidirectional associations, the following hypotheses were advanced tentatively while leaning on prevailing theorizing and selected findings: (1) Passive social media usage will predict higher subsequent negative emotions, whereas active usage will predict lower subsequent negative emotions (Kross et al., 2013; Schmuck et al., 2019) and (2) higher negative emotions will predict subsequent active usage (reflecting posting as a coping mechanism; Frison & Eggermont, 2017; Puukko et al., 2020).

Subjective experiences of emotions and social media usage vary considerably between individuals; what is considered an extremely bothersome negative emotion for one person may be considered routine and manageable for another (Wylie et al., 2023). We thus focused on within- rather than between-person associations in our analyses. For within-person analyses, each participant’s own, typical level of negative emotions/social media usage across multiple measurements (rather than the levels of other participants) serves as the reference point for whether their negative emotions/social media usage at any single measurement point is considered high or low.

## Method

### Participants

Adolescents (*N* = 154; 40% female; *M*_*age*_ = 13.47 years, *SD* = 0.58, Range = 12–15 [95% 13–14]; 88% Caucasian, 4% First Nations, 3% Asian, 1% Black, and 4% multiethnic/other) were participating in the third wave of a 5-year annual longitudinal study and were e-mailed about the additional opportunity to participate in the current experience sampling study. They were initially recruited through the Queen’s University Developmental Database, which consists of local families in Southern Ontario, Canada, who had previously consented at community events to be contacted should they be eligible to participate in any studies. This database is maintained by faculty and graduate students at the Department of Psychology, Queen’s University. Families’ household incomes (CAD) were reported as 9% < 50,000, 13% 50,000–75,000, 18% 75,000–100,000, 29% 100,000–150,000, and 31% > 150,000. For the current study, adolescents were prompted to provide data four times per day over 14 days (i.e., up to 8,624 prompts at level 1). This level-1 sample size provided sufficient power to detect at least a small-moderate effect in a multilevel analysis framework with the present study’s average estimated intraclass correlation (ICC; see Results section for obtained ICCs and Kleiman, n.d. for power analysis tool).

### Procedure

Parental consent and adolescent assent were collected each year of the longitudinal study and specifically for the experience sampling portion, which ran from October 2020 to March 2021. Participants completed a baseline online questionnaire on their demographics and social-emotional functioning. Adolescents then downloaded the MetricWire experience sampling smartphone app (MetricWire, Kitchener, Canada) on their personal digital device. Starting a day later, they were prompted by the app to answer questions at 11:00 a.m., 2:15 p.m., 5:30 p.m., and 8:45 p.m. every day for 14 days (56 prompts). Each prompt took approximately 2–3 min to complete, and participants were given a 90-min completion window to accommodate their varying schedules. Research assistants monitored the fidelity of prompt completion each evening and sent reminder e-mails to those who missed any prompts. At the end of the 14-day period, parents were debriefed by email and e-transferred up to $86 for their child’s participation ($30 for the baseline survey and $1 for each of the 56 prompts completed). They also received a ticket towards a raffle to be conducted at the end of the longitudinal study.

### Measures

Each prompt included a brief set of questions probing the following elements (in order): (a) current mood, (b) digital device usage, (c) most bothersome negative emotion experienced since the last prompt (anger, sadness, anxiety, or embarrassment; forced choice), (d) intensity of the emotion, (e) activities attributed to generating the emotion, (f) social surroundings, (g) digital and in-person support sought and received for the emotion, (h) response to the emotion (i.e., regulation strategy), and (i) perceived success in managing the emotion. The present analysis focused on elements (b) digital device usage and (d) intensity of the [most bothersome negative] emotion.

#### Browsing and Posting

To probe their digital device usage, participants were asked, “Since the last prompt, in which of the following did you engage (select all that apply): Browsing social media, posting on social media, messaging/communicating (texting, e-mailing, calling, etc.), solo video game, multiplayer video game.” Given the present study’s focus on passive and active subtypes of social media usage, separate binary variables were created for browsing social media and posting on social media at each prompt (0 = *no browsing*, 1 = *browsing*, 0 = *no posting*, 1 = *posting*, respectively). Since participants could select all digital activities that applied, it was possible for them to indicate browsing *and* posting for the same prompt. Browsing occurred 3,837 times (i.e., 62% of 6,218 valid prompts) and co-occurred with posting 25% of the time. To isolate browsing, shared instances of browsing and posting were coded as missing for the browsing variable, and resulting incidences of “pure” browsing (55% of 5,260 valid prompts) were used in analyses. Posting was relatively less prevalent overall, occurring 1,004 times (i.e., 16% of 6,212 valid prompts; see Table 1), and it overlapped with browsing 95% of the time. Thus, to maintain sufficient variability in posting for analyses, any prompt including posting was coded as 1 for the posting variable regardless of whether it co-occurred with browsing during the same reporting window.

#### Negative Emotions

Momentary negative emotions were measured as a dimension of mental health. For the most bothersome negative emotion (anger, sadness, anxiety, or embarrassment; forced choice) experienced since their last prompt, participants were asked, “How intensely did you experience [selected emotion]” on a 10-point scale from 1 (*not intense at all, I barely noticed it*) to 10 (*the most intense*). Anxiety was most commonly reported (41%), followed by sadness (27%), anger (22%), and embarrassment (10%).

### Data Analysis Plan

Analyses were conducted using M*plus* 8.10 (Muthén & Muthén, 1998–2017). First, missing data were inspected. Descriptive analyses and within/between correlations were then conducted. For the main analyses, multilevel modelling (Raudenbush & Bryk, 2002) was used to account for the nested data structure with up to 56 prompts belonging to the same adolescent. Repeated measures at the within level (i.e., level 1; varying across prompts within adolescents) were browsing, posting, and negative emotions. Covariates at the between level (i.e., level 2; fixed within adolescents but differing between them) were continuous age in years and gender (0 = *female*, 1 = *male*). Level-1 predictors were group-mean centered (also referred to as person-mean centering or centering within context). At level 2, age was grand-mean centered whereas gender was left uncentered because it already had a meaningful zero point.

Four two-level multilevel models were conducted to assess the bidirectional predictive associations between browsing/posting and negative emotions. All four models were lagged such that the outcome at time *T* + *1* was regressed on level-1 predictors at time *T*. Initial levels of the outcome at time *T* were also included as a level-1 covariate to test Granger causality, a quasi-causal test of directionality that accounts for bias stemming from moment-to-moment stability in the outcome (see Hamaker & Wichers, 2017). The effect of passing time over 14 days of study was not of theoretical interest. Time—coded 0 (first prompt) to 55 (last prompt)—was thus included as a level-1 covariate to detrend the outcomes and avoid temporal biasing of the level-1 associations (Wang & Maxwell, 2015). All models used Bayesian estimation because it produces smaller standard errors and fewer convergence issues with categorical and/or non-normally distributed data from samples of similar size to the current study (Muthén et al., 2015). The WAMBS-Checklist was followed to rule out common issues in Bayesian analyses (e.g., instability of findings across increased iterations, high degrees of autocorrelation; see Depaoli and van de Schoot, 2017).

**Fig. 1:**
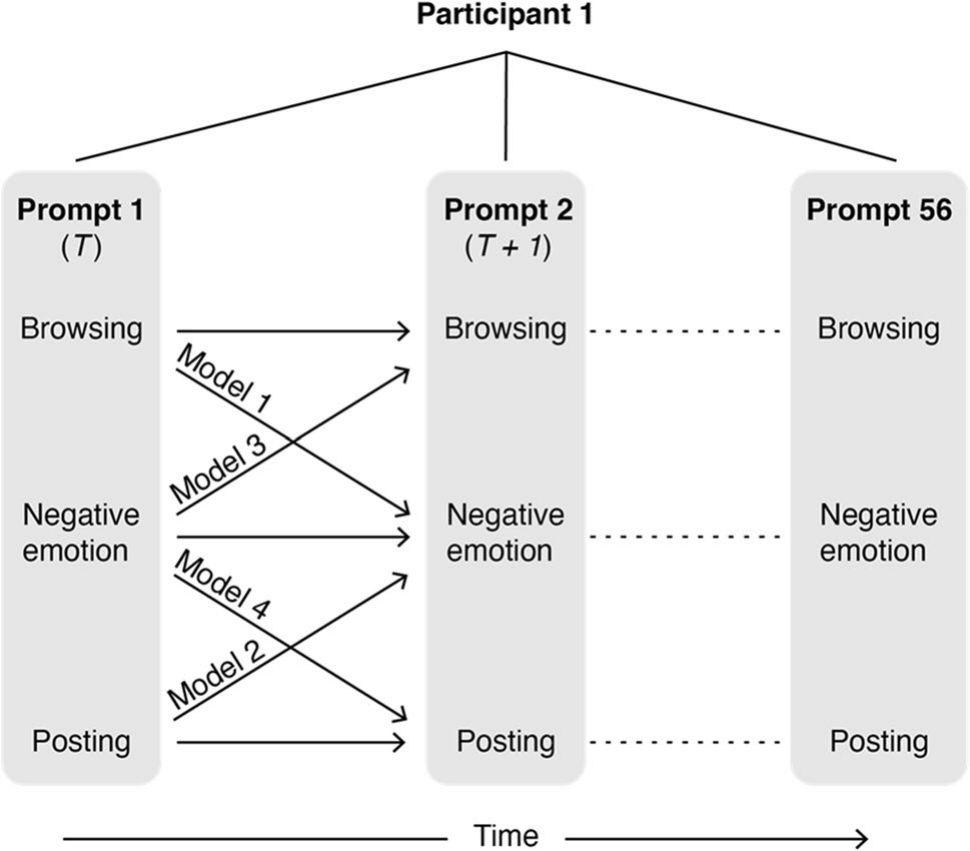
Models testing bidirectional associations of browsing/posting and negative emotions across 56 time points within each participant

As depicted in Fig. 1, the four core lagged models tested whether browsing/posting at a given prompt predicted negative emotions a few hours later at the next prompt, and vice versa. Model 1 tested whether browsing predicted subsequent negative emotions. Model 2 tested whether posting predicted subsequent negative emotions. Model 3 tested whether negative emotions predicted subsequent browsing. Model 4 tested whether negative emotions predicted subsequent posting. Browsing and posting were specified as categorical dependent variables in Models 3 and 4, respectively; hence, probit regression was used for these models.

Each of the four lagged models was run two more times to assess the reproducibility of the findings. First, the models were re-run while estimating missing data to make full use of the potential level-1 sample size assuming no attrition (i.e., *N* = 8624). Second, the models were re-run excluding the outcomes’ morning prompts to account for potential bias from the disproportionate overnight time lag between the last prompt of one day and the first prompt of the next day. Finally, supplemental models testing concurrent (i.e., same prompt) associations were conducted to understand the relative importance of lagging the outcome for the significance and strength of the hypothesized associations. For those interested in comparing within- and between-level effects, the concurrent models further tested between-level associations of cluster-averaged browsing/posting on overall negative emotions across the study.

## Results

Adolescents provided at least partial data for 6,240 (72%) of 8,624 possible prompts, which is comparable to the compliance rates of similar experience sampling studies (e.g., Beyens et al., 2021; Kross et al., 2013). Missing data in the supplemental models were handled with full information maximum likelihood (FIML) estimation (see Enders, 2022). Descriptive statistics and zero-order within and between correlations are reported in Table 1. Notably, at the within level, browsing and posting tended to co-occur with elevated negative emotions. At the between level, adolescents who browsed and posted more across the 14 days tended to have higher negative emotions across the same period relative to adolescents who browsed and posted less (also see Table S3 for between-level effects controlling for age and gender). Older adolescents browsed more than younger adolescents. Across all 14 days, girls had higher negative emotions and browsed more relative to boys. The negative within-level browsing–posting correlation should be regarded as spurious because, as per above, browsing was recoded to remove same-prompt occurrences with posting.

**Table 1:** Descriptive statistics and zero-order correlations at the within and between levels

Variable	1	2	3	4	5	*M*	*SD*	Observed range
1. Negative emotions	1	.42^***^	.34^***^	.08	− .25^**^	3.21	2.48	1–10
2. Browsing	.08^**^	1	.31^***^	.31^***^	− .19^*^	.55	.50	0–1
3. Posting	.14^**^	− .36^***^	1	.04	.03	.16	.37	0–1
4. Age	—	—	—	1	− .13	13.47	.58	12–15
5. Gender	—	—	—		1	—	—	—

Left side = within correlations. Right side = between correlations (within-only variables were cluster-mean averaged across 56 prompts). Age in continuous years. Gender (0 = *female*, 1 = *male*). ^*^p < .05. ^**^p < .01. ^***^p < .001

Null models for negative emotions, browsing, and posting revealed ICCs of 0.38, 0.43, and 0.50, respectively. In other words, up to 38%, 43%, and 50% of the variance in negative emotions, browsing, and posting, respectively, could be explained by differences between adolescents. These amounts were sufficient to justify the use of multilevel modelling and aligned with typical ICCs reported for intensive self-reported longitudinal studies (Bolger & Laurenceau, 2013).

The results of the final lagged models are reported in Table 2. For Model 1 testing whether browsing predicted subsequent negative emotions, prompts with browsing were associated with higher-than-usual negative emotions at the subsequent prompt, above and beyond the positive autoregressive effect of adolescents’ negative emotions at the prior prompt. As per Model 2, this finding did not replicate with posting in place of browsing as a predictor of subsequent negative emotions. Thus, hypothesis 1 was partially confirmed because browsing predicted higher negative emotions but posting did not predict lower negative emotions. Model 3 results indicated that higher-than-usual negative emotions did not significantly predict the presence of browsing at the following prompt. Model 4 results revealed that higher-than-usual negative emotions did not predict the presence of posting at the subsequent prompt, rejecting hypothesis 2.

**Table 2:** Results of lagged multilevel models testing bidirectional associations of negative emotions, browsing, and posting

Model and outcome	Predictor	*β*	Posterior *SD*	*p*	95% CI
Model 1: negative emotions (*T* + *1*)	Browsing (*T*)	0.11	0.04	.002	[0.04, 0.19]
PPP = .51	Negative emotions (*T*)	0.18	0.01	< .001	[0.15, 0.21]
*R*^2^ within = .04, *p* < .001	Time	0.05	0.02	< .001	[0.02, 0.08]
*R*^2^ between = .10, *p* < .001	Age	0.07	0.08	.20	[− 0.09, 0.23]
	Gender	− 0.57	0.15	.001	[− 0.86, − 0.25]
Model 2: negative emotions (*T* + *1*)	Posting (*T*)	0.002	0.05	.49	[− 0.10, 0.11]
PPP = .51	Negative emotions (*T*)	0.18	0.01	< .001	[0.16, 0.21]
*R*^2^ within = .04, *p* < .001	Time	0.04	0.01	.002	[0.01, 0.07]
*R*^2^ between = .08, *p* < .001	Age	0.07	0.08	.19	[− 0.09, 0.23]
	Gender	− 0.48	0.16	.003	[− 0.77, − 0.15]
Model 3: browsing (*T* + *1*)	Negative emotions (*T*)	− 0.02	0.02	.15	[− 0.07, 0.02]
PPP = .50	Browsing (*T*)	0.35	0.05	< .001	[0.24, 0.45]
*R*^2^ within = .05, *p* < .001	Time	− 0.15	0.02	< .001	[− 0.19, − 0.11]
*R*^2^ between = .12, *p* < .001	Age	0.28	0.08	< .001	[0.12, 0.43]
	Gender	− 0.33	0.16	.028	[− 0.63, 0.004]
Model 4: posting (*T* + *1*)	Negative emotions (*T*)	0.03	0.03	.15	[− 0.03, 0.08]
PPP = .49	Posting (*T*)	0.63	0.07	< .001	[0.48, 0.77]
*R*^2^ within = .03, *p* < .001	Time	− 0.05	0.03	.053	[− 0.12, 0.01]
*R*^2^ between = .02, *p* < .001	Age	0.09	0.09	.16	[− 0.09, 0.26]
	Gender	− 0.08	0.18	.34	[− 0.43, 0.27]

*PPP* posterior predictive *p*-value. *T* + *1* lagged variable/subsequent levels (all predictors assessed as *T* (i.e., prior levels)). *Time* prompt number ranging from 0 (first prompt) to 55 (last prompt). *CI* confidence interval. One-tailed Bayesian *p*-value reported

The significant lagged effect of browsing was relatively small (*β* = 0.11) and explained a small amount of within-level variance in negative emotions alongside other level-1 predictors (4%). By comparison, a considerably larger aggregate-level correlation between negative emotions and browsing manifested at the between level (*r* = .42; Table 1), although the between-level effect of browsing diminished when age and gender were included as covariates (Table S3). There were consistent effects of gender across Models 1–3, such that girls experienced higher levels of negative emotions and engaged in (marginally) more browsing across the 14 days of study. Only one direct effect of age emerged, as older adolescents were more likely to browse. For all models, prior levels/occurrence of the outcome significantly and positively predicted subsequent levels/occurrence of the outcome. There was an average increase in negative emotions, a decrease in browsing, and no mean-level change in posting across the 2 weeks—results held after controlling for these within-level temporal trends.

As depicted in Table S1 of the Online Supplementary Material, all effects were consistent when FIML was used to estimate the missing data. Similarly, as per Table S2, the results fully replicated when time lags between prompts were equalized by removing morning prompts for the outcomes. The effects in Table 2 can thus be interpreted as robust to attrition and differing time lags. Finally, further speaking to the importance of considering lagged associations between social media use and momentary mental health indicators, the effect of browsing on negative emotions did not replicate within the same prompt (Table S3).

## Discussion

The current study expanded the empirical literature by testing the bidirectional effects of momentary browsing vs. posting on negative emotions in early adolescence. As hypothesized, browsing predicted higher negative emotions hours later. This short-term longitudinal finding aligns with previous cross-sectional (e.g., Thorisdottir et al., 2019) and long-term longitudinal (Frison & Eggermont, 2017; Shakya & Christakis, 2017) studies implicating browsing in adolescents’ mental health challenges. A commonly cited explanation for these findings is that passively browsing others’ curated content increases upward social comparisons and envy (Wang et al., 2019). Indeed, adolescents who browse more on social media are envy prone (Scherr et al., 2019), and Valkenburg, Beyens et al. (2022) linked browsing-induced envy to lower happiness within a lagged experience sampling design. An alternative explanation may be time wasting. Adolescents engaged in browsing for well over half of the 6,218 snapshots provided in this study. Such a significant amount of time on social media likely meant less time for other daily matters, which could have resulted in time pressure, guilt, and related negative emotions (e.g., worrying there is not enough time to do all important things). This theory has yet to be tested directly, but some evidence supports its plausibility as social media has been linked to guilt proneness (Panek, 2014) and inconsistent sleep (Hamilton et al., 2020).

Posting was hypothesized to alleviate negative emotions via social engagement, support, and related positive emotions (Schmuck et al., 2019), but it was not associated with subsequent negative emotions. This null finding implies variability in adolescents’ emotional responses to posting. If one post is received with attentive support but another post receives little attention or even negative attention, the emotional effects of such posts may cancel each other out, resulting in a null combined within-person effect. Indeed, both positive and negative effects of posting on emotions have been demonstrated at the between level (i.e., between adolescents) depending on their social network quality (Selfhout et al., 2009) and post content (Pellicane et al., 2021).

Regarding the opposite directional effect, higher-than-usual negative emotions did not predict subsequent browsing (for a similar finding, see Steinsbekk et al., 2023). Mood management theory argues that an individual’s choice of media depends on whether they want to induce/preserve a positive mood or reduce a negative one (Zillmann, 2000). However, adolescents in the present study did not reliably choose browsing as a means of managing negative emotions. They may have recognized previous instances where negative emotions stemmed from browsing, opting not to browse more and risk amplifying their already negative emotional state. If not initial negative emotions, the question remains as to what else triggers the process of browsing-induced negative emotions. Future studies could consider the possibility that emotionally harmful browsing sessions emerge randomly and innocently rather than in response to a negative state (e.g., as a sequence of checking social media at random, unexpectedly binging, falling behind on homework, and experiencing anxiety).

Past studies have suggested posting as a coping response to negative emotions (Frison & Eggermont, 2017; Puukko et al., 2020). In the present study, experiencing negative emotions did not predict posting hours later. Hypothesis 2 advancing posting as a *subsequent* coping response to negative emotions must therefore be rejected based on the current findings. Different forms of “cope-posting” may have varying effects on subsequent negative emotions (e.g., venting vs. sensitively requesting emotional support) and could be explored in future studies.

Overall, the current short-term longitudinal study yielded clearer and more theoretically aligned findings than long-term longitudinal studies assessing the same directional effects. Researchers rarely justify their choice of time interval for longitudinal studies, despite timing often affecting whether the same hypothesized effect is present or not (Timmons & Preacher, 2015). Long-term longitudinal studies spanning months or years are susceptible to intermediary confounds (Gollob & Reichardt, 1987). Adolescents experience significant developmental shifts in emotions and relationships across even 1 year (Hollenstein & Lougheed, 2013). These shifts could have interim effects on both social media usage and mental health, obfuscating any would-be annual associations between these constructs. Long-term longitudinal studies also tend to rely on self-reports of trait-level or “typical” social media usage. Adolescents may inaccurately recall their typical social media usage across large periods of time because experiences on social media are interwoven with daily life and difficult to distinguish from other activities (Sewall et al., 2020; Verbeij et al., 2021). The present findings affirm these long-term drawbacks and underscore the utility of short-term, intensive developmental designs for emerging research on this topic.

Early adolescence marks the first stages of identity formation and social media use (Allison & Schultz, 2001; Spies Shapiro & Margolin, 2014). The current participants’ age may thus explain the significant negative effects of browsing as older adolescents may be, on the whole, more experienced and developmentally prepared to avoid and/or cope with the affective pitfalls of passive social media use (Hollenstein & Colasante, 2020). Empirically, the current findings align with Orben et al. (2022), who, at a much broader level with over 80,000 participants spanning 10–80 years of age, isolated early adolescence as the period with the strongest negative correlations between social media use and life satisfaction. The few studies most similar to the present study in design and age range under study yielded mixed negative and null effects of browsing (Beyens et al., 2021; Valkenburg, Beyens et al., 2022), although there are many design differences between these studies and the current one (e.g., different outcome measures and measurement frequencies). Future studies should explore age-related buffering capacities by directly comparing younger and older cohorts of adolescents within the same design frameworks. Repeating short-term longitudinal experiments within a long-term longitudinal study would be even more favorable. Overall, the current findings tentatively build on evidence for a “sensitive window of social media challenges” in early adolescence.

Some limitations of the current study should be considered. The core effect of browsing was small, and its practical significance should be interpreted accordingly. Indirect and moderating effects should be explored to understand when social media–mental health effects are larger (or smaller). Despite its advantages of ecological validity, the present study was restricted to Granger causality. Future experimental studies are needed to rule out confounds. Active social media usage heavily overlapped with passive usage (also see Beyens et al., 2020), making it difficult to discern the specific effects of the former. In reality, posting is often part and parcel of browsing (i.e., browsing content induces a posting response from the adolescent); unprompted content creation is less common. Future studies interested in parsing the effects of active vs. passive use may wish to measure each on a continuous scale to compare them on the basis of relative intensity rather than occurrence. Moreover, broadly measuring “browsing” and “posting” may mask important heterogeneity in passive vs. active social media use—utilizing measures of even more specific behaviors may yield more insight into the mechanisms behind social media–mental health links in early adolescence. Also, although prompting adolescents to respond multiple times per day reduces the risk of recall bias, it has been shown that self-reports are not always a reliable indication of actual social media use. Future studies should incorporate real-time tracking of actual social media use (noting potential ethical/privacy implications; for a discussion, see Schønning et al., 2020). Finally, the present study solely examined negative emotions as one of many momentary dimensions of mental health. Future studies should strive for a holistic assessment of negative and positive indicators of affective well-being and mental health.

To conclude, the present findings suggest that interrelations of social media use and mental health depend on timing (with negative effects emerging over subsequent hours rather than concurrently), type (with negative effects emerging for browsing but not posting), and development (with negative effects emerging in a sample of early adolescents). Browsing may be the type of social media use most likely to induce negative affective experiences in early adolescence, but the overall evidence linking social media usage to negative emotions in the present study was weak.

## Supplementary Information

Below is the link to the electronic supplementary material.

Supplementary file1 (DOCX 25 KB)

## Additional Information

### Data Availability

The data underlying this article is available via the following link: https://osf.io/myajt/?view_only=e8455dc5da474615b2a617d00283db7e.
