# Supplementary material for: Bidirectional Associations of Adolescents’ Momentary Social Media Use and Negative Emotions
Source: Affect Sci. 2024 Jun 26;5(4):300–9. doi: 10.1007/s42761-024-00244-2 (PMC11624169; doi:10.1007/s42761-024-00244-2)
Supplement: Supplementary file 1 — Supplementary file1 (DOCX 25 KB) [file 42761_2024_244_MOESM1_ESM.docx]

**Online Supplementary Material**

**Table S1**

*Results of Lagged Multilevel Models with Missing Data Estimated*

| Model and Outcome | Predictor | β | Posterior *SD* | *p* | 95% CI |
| --- | --- | --- | --- | --- | --- |
| Model 1: Negative emotions (*T+1*) | Browsing | 0.04 | 0.02 | .004 | [0.01, 0.07] |
| PPP = .51 | Negative emotions (*T*) | 0.19 | 0.01 | <.001 | [0.16, 0.22] |
| *R*^2^ within = .04, *p* < .001 | Time | 0.05 | 0.01 | <.001 | [0.03, 0.08] |
| *R*^2^ between = .09, *p* < .001 | Age | 0.07 | 0.08 | .21 | [-.09, 0.22] |
|  | Gender | -0.53 | 0.15 | <.001 | [-0.82, -0.22] |
| Model 2: Negative emotions (*T+1*) | Posting | 0.001 | 0.01 | .47 | [-0.03, 0.03] |
| PPP = .48 | Negative emotions (*T*) | 0.19 | 0.01 | <.001 | [0.16, 0.22] |
| *R*^2^ within = .04, *p* < .001 | Time | 0.05 | 0.01 | <.001 | [0.02, 0.07] |
| *R*^2^ between = .08, *p* < .001 | Age | 0.06 | 0.08 | .22 | [-0.09, 0.22] |
|  | Gender | -0.53 | 0.15 | .001 | [-0.81, -0.22] |
| Model 3: Browsing (*T+1*) | Negative Emotions | -0.01 | 0.02 | .28 | [-0.06, 0.03] |
| PPP = .47 | Browsing (*T*) | 0.13 | 0.02 | <.001 | [0.09, 0.17] |
| *R*^2^ within = .04, *p* < .001 | Time | -0.14 | 0.02 | <.001 | [-0.18, -0.10] |
| *R*^2^ between = .12, *p* < .001 | Age | 0.30 | 0.07 | .005 | [0.15, 0.44] |
|  | Gender | -0.24 | 0.16 | .06 | [-0.54, 0.07] |
| Model 4: Posting (*T+1*) | Negative Emotions | 0.03 | 0.03 | .15 | [-0.02, 0.08] |
| PPP = .49 | Posting (*T*) | 0.16 | 0.02 | <.001 | [0.13, 0.20] |
| *R*^2^ within = .03, *p* < .001 | Time | -0.03 | 0.03 | .15 | [-0.08, 0.02] |
| *R*^2^ between = .02, *p* < .001 | Age | 0.10 | 0.09 | .12 | [-0.07, 0.27] |
|  | Gender | -0.08 | 0.17 | .33 | [-0.41, 0.26] |

*Note.* PPP = posterior predictive *p*-value. *T + 1* = lagged variable/subsequent levels (all predictors assessed as *T* [i.e., prior levels]). Time = prompt number ranging from 0 (first prompt) to 55 (last prompt). CI = confidence interval. One-tailed Bayesian *p*-value reported. FIML in M*plus* forces STDYX coefficients for binary predictors, giving the appearance of a smaller browsing effect.

**Table S2**

*Results of Lagged Multilevel Models with Morning Prompts Omitted*

| Model and Outcome | Predictor | β | Posterior *SD* | *p* | 95% CI |
| --- | --- | --- | --- | --- | --- |
| Model 1: Negative emotions (*T+1*) | Browsing (*T*) | 0.13 | 0.05 | .002 | [0.04, 0.23] |
| PPP = .53 | Negative emotions (*T*) | 0.18 | 0.02 | <.001 | [0.14, 0.21] |
| *R*^2^ within = .04, *p* < .001 | Time | 0.04 | 0.02 | .02 | [0.004, 0.07] |
| *R*^2^ between = .10, *p* < .001 | Age | 0.09 | 0.08 | .14 | [-.07, 0.25] |
|  | Gender | -0.55 | 0.16 | <.001 | [-0.85, -0.22] |
| Model 2: Negative emotions (*T+1*) | Posting (*T*) | 0.02 | 0.06 | .35 | [-0.10, 0.15] |
| PPP = .48 | Negative emotions (*T*) | 0.18 | 0.02 | <.001 | [0.15, 0.21] |
| *R*^2^ within = .04, *p* < .001 | Time | 0.03 | 0.02 | .026 | [0.0001, 0.06] |
| *R*^2^ between = .07, *p* < .001 | Age | 0.09 | 0.08 | .13 | [-0.07, 0.25] |
|  | Gender | -0.45 | 0.16 | .004 | [-0.74, -0.12] |
| Model 3: Browsing (*T+1*) | Negative Emotions (*T*) | -0.02 | 0.03 | .24 | [-0.07, 0.03] |
| PPP = .48 | Browsing (*T*) | 0.28 | 0.07 | <.001 | [0.15, 0.41] |
| *R*^2^ within = .05, *p* < .001 | Time | -0.18 | 0.03 | <.001 | [-0.24, -0.13] |
| *R*^2^ between = .16, *p* < .001 | Age | 0.28 | 0.08 | <.001 | [0.12, 0.43] |
|  | Gender | -0.46 | 0.16 | .004 | [-0.76, -0.12] |
| Model 4: Posting (*T+1*) | Negative Emotions (*T*) | 0.03 | 0.03 | .17 | [-0.03, 0.09] |
| PPP = .48 | Posting (*T*) | 0.65 | 0.08 | <.001 | [0.48, 0.80] |
| *R*^2^ within = .03, *p* < .001 | Time | -0.04 | 0.03 | .11 | [-0.11, 0.02] |
| *R*^2^ between = .01, *p* < .001 | Age | 0.02 | 0.05 | .49 | [-0.10, 0.11] |
|  | Gender | -0.14 | 0.18 | .21 | [-0.49, 0.21] |

*Note.* PPP = posterior predictive *p*-value. *T + 1* = lagged variable/subsequent levels (all predictors assessed as *T* [i.e., prior levels]). Time = prompt number ranging from 0 (first prompt) to 55 (last prompt). CI = confidence interval. One-tailed Bayesian *p*-value reported.

**Table S3**

*Results of Concurrent Multilevel Models Including Within- and Between-Level Associations of Browsing and Posting*

| Outcome | Predictor | β | Posterior *SD* | *p* | 95% CI |
| --- | --- | --- | --- | --- | --- |
| Negative emotions | Browsing (L1) | -0.01 | 0.03 | .32 | [-0.06, 0.04] |
| PPP = .51 | Time | 0.06 | 0.01 | <.001 | [0.04, 0.08] |
| *R*^2^ within = .004, *p* < .001 | Age | 0.03 | 0.06 | .35 | [-0.10, 0.15] |
| *R*^2^ between = .06, *p* < .001 | Gender | -0.36 | 0.13 | .004 | [-0.62, -0.10] |
|  | Browsing (L2) | 0.10 | 0.06 | .054 | [-0.02, 0.21] |
| Negative emotions | Posting (L1) | 0.05 | 0.03 | .07 | [-0.02, 0.12] |
| PPP = .48 | Time | 0.06 | 0.01 | <.001 | [0.04, 0.08] |
| *R*^2^ within = .004, *p* < .001 | Age | 0.05 | 0.06 | .19 | [-0.07, 0.17] |
| *R*^2^ between = .11, *p* < .001 | Gender | -0.33 | 0.13 | .004 | [-0.57, -0.08] |
|  | Posting (L2) | 0.29 | 0.06 | <.001 | [0.17, 0.39] |

*Note.* L1 = level-1 within-person association. L2 = level-2 between-person association. PPP = posterior predictive *p*-value. Time = prompt number ranging from 0 (first prompt) to 55 (last prompt). CI = confidence interval. One-tailed Bayesian *p*-value reported.
